# Complete Genome Sequence of *Brachybacterium* sp. Strain NBEC-018, Isolated from Nematode-Infected Potatoes

**DOI:** 10.1128/mra.00966-22

**Published:** 2022-11-17

**Authors:** Ling Chen, Yueying Wang, Nanxi Liu, Lei Zhu, Yong Min, Yimin Qiu, Yuxi Tian, Xiaoyan Liu

**Affiliations:** a National Biopesticide Engineering Technology Research Center, Hubei Biopesticide Engineering Research Center, Hubei Academy of Agricultural Sciences, Biopesticide Branch of the Hubei Innovation Center of Agricultural Science and Technology, Wuhan, People’s Republic of China; University of Maryland School of Medicine

## Abstract

*Brachybacterium* species are ubiquitous Gram-positive bacteria. Here, we report the complete genome sequence of *Brachybacterium* sp. strain NBEC-018. The strain was isolated from rotten potatoes that were infected with potato rot nematodes (Ditylenchus destructor). The genome sequence will be beneficial to clarify the ecological role of *Brachybacterium* species.

## ANNOUNCEMENT

The genus *Brachybacterium*, which belongs to the family *Dermabacteraceae*, was first described by Collins et al. in 1988 (1). Members of this genus are mainly characterized as Gram-stain-positive bacteria that have coccus-shaped cells (2). *Brachybacterium* species have been isolated from various habitats (3). Here, we report the complete genome sequence of *Brachybacterium* sp. strain NBEC-018, which was isolated from nematode-infected potatoes. The genomic analysis of this strain will be beneficial to clarify the ecological role of *Brachybacterium* species.

Strain NBEC-018 was isolated from a sample of rotten potatoes (Solanum tuberosum) collected in a farm field in Wuhan, China (30.62N, 114.13E). The collected potatoes were infected with potato rot nematodes (Ditylenchus destructor), an important plant-parasitic nematode (4). After the nematode-infected wounds on the potatoes were washed with sterile water, a bacterial suspension was obtained. The bacteria suspended in the water were spread on Luria-Bertani (LB) agar and incubated overnight at 30°C. Colonies with different bacterial morphology were selected for genomic sequencing. Strain NBEC-018 was passaged 15 times on LB agar and then incubated overnight in LB broth at 30°C with shaking at 220 rpm. The DNA of NBEC-018 was extracted by using the blood and cell culture DNA kit (Qiagen, Germany) following the manufacturer’s instructions. Genome sequencing was carried out using a combination of short-read sequencing and long-read sequencing. For short-read sequencing, genomic DNA was fragmented with an LE220 focused ultrasonicator (Covaris, USA), and then DNA fragments with an average size of 200 to 400 bp were selected by using AMPure XP magnetic beads (Beckman, Germany). A paired-end short-read sequencing library was prepared using the MGIEasy FS PCR-free library preparation set (MGI Tech Co., Ltd.). The sequencing was performed on a DNBSEQ-T7 instrument (MGI, China) using 150-nucleotide-long reads. Raw reads were trimmed by removing low-quality reads and adapters using the FASTQ preprocessing program fastp v.0.23.2 (5) with default settings. The resulting short-read data contained 7,137,506 short reads, with a total length of 1,070,546,096 bp. For long-read sequencing, the same genomic DNA preparation was used directly to construct a DNA library using a ligation sequencing kit (SQK-LSK-109; Oxford Nanopore Technologies, Ltd. [ONT], Oxford, UK) without DNA shearing. The library was sequenced using a PromethION sequencer (ONT) with an R9.4.1 flow cell (FLO-MIN106). Base calling, barcode segmentation, and adapter sequence removal from the raw sequences were then performed using Guppy v.4.4.2 (6) with default settings. A total of 120,706 reads, with an *N*_50_ value of 30,331 bp, were obtained.

By using Unicycler v.0.4.8 (7) with default settings, the two sequencing data sets were assembled into a complete circular chromosome. The genome size of NBEC-018 is 3,863,999 bp (GC content, 73.24%), with genome coverage of 709×. The genome of NBEC-018 has an average nucleotide identity (ANI) (as determined using the ANI Calculator [https://www.ezbiocloud.net/tools/ani]) of 96.22% with *Brachybacterium* sp. strain SGAir0954 (GenBank accession number CP027295) (3); therefore, NBEC-018 was identified as a *Brachybacterium* species isolate. The genome of NBEC-018 contains 3,467 coding sequences (CDSs), 53 tRNA genes, and 9 rRNA genes, as annotated by NCBI PGAP v.6.1 (8).

### Data availability.

The genome sequence of *Brachybacterium* sp. strain NBEC-018 has been deposited in the GenBank database under accession number CP100650. Sequencing reads have been submitted to the SRA database under accession numbers SRR19995284 and SRR19995286.
